# Exploring why quality circles work in primary health care: a realist review protocol

**DOI:** 10.1186/2046-4053-2-110

**Published:** 2013-12-09

**Authors:** Adrian Rohrbasser, Sharon Mickan, Janet Harris

**Affiliations:** 1 santémed Health Care Centre, Friedtalweg 18, Wil 9500, Switzerland; 2 Department of Primary Health Care Sciences, University of Oxford, New Radcliffe House, Walton Street, Jericho, Oxford OX2 6NW, UK; 3 Section of Public Health, School of Health and Related Research (ScHARR), University of Sheffield, Regent Court, 30 Regent Street, Sheffield S1 4DA, UK

**Keywords:** Realist synthesis, Realist review, Quality circle, Peer review group, Small group work, Quality improvement

## Abstract

**Background:**

Quality circles (QCs) are commonly used in primary health care in Europe to consider and improve standard practice over time. They represent a complex social intervention that occurs within the fast-changing system of primary health care. Numerous controlled trials, reviews, and studies have shown small but unpredictable positive effect sizes on behavior change. Although QCs seem to be effective, stakeholders have difficulty understanding how the results are achieved and in generalizing the results with confidence. They also lack understanding of the active components of QCs which result in changes in the behavior of health care professionals. This protocol for a realist synthesis will examine how configurations of components and the contextual features of QCs influence their performance.

**Methods/Design:**

Stakeholder interviews and a scoping search revealed the processes of QCs and helped to describe their core components and underlying theories. After clarifying their historical and geographical distribution, a purposive and systematic search was developed to identify relevant papers to answer the research questions, which are: understanding why, how, and when QCs work, over what time frame, and in what circumstances. After selecting and abstracting appropriate data, configurations of contexts and mechanisms which influence the outcome of QCs within each study will be identified. Studies will be grouped by similar propositional statements in order to identify patterns and validation from stakeholders sought. Finally, theories will be explored in order to explain these patterns and to help stakeholders maintain and improve QC performance.

**Discussion:**

Analyzing context-mechanism-outcome (CMO) patterns will reveal how QCs work and how contextual factors interact to influence their outcome. The aim is to investigate unique configurations that enable them to improve the performance of health care professionals. Using a standardized reporting system, this realist review will allow the research questions to be answered to the satisfaction of key stakeholders and enable on-going critical examination and dissemination of the findings.

**Study registration:**

PROSPERO registration number: CRD42013004826.

## Background

### Rationale for the review

Quality circles (QCs) are small groups of 6 to 12 professionals from a similar background who meet at regular intervals to discuss and review their clinical practice. The focus of discussion is usually a critical evaluation of a key issue related to quality in health care. QCs select the issues they want to deal with themselves, decide on their method of gathering data, and determine a way of finding solutions to prioritized problems. Facilitators observe and lead the group through the circle of quality improvement [1-8]. The main purposes of QCs in primary health care are continuing professional development (CPD), quality improvement, and continuing medical education (CME) [3,9-13].

QCs have been established predominantly in Germany, Switzerland, Austria, Sweden, Denmark, Norway, Belgium, France, the Netherlands, and Ireland [14]. In Switzerland, they have been established as the main method of quality improvement and CPD and, currently, 80% of all physicians working in primary health care regularly attend QCs [15].

Numerous studies suggest that QCs improve individual and group performance in terms of costs, ordering of tests, prescription habits, and adherence to clinical practice guidelines, thus resulting in better patient outcome measures and changes in performance indicators [6,8,16-22]. Several systematic reviews (SRs) in the Cochrane Library show that elements of QCs such as educational materials, workshops, audit and feedback (with or without outreach visits), and local knowledge experts have a positive impact on the behavior of practitioners [23-27]. Facilitation is a key factor that has been shown in several reviews to be effective [28,29]. Although systematic reviews of QCs provide summaries of their effectiveness, they are based on the assumption that the intervention has causal powers, and are typically unable to explain considerable variations in effectiveness because the original trials that are included in the review rarely explore the influence of surrounding contexts effectiveness [30].

Stakeholders in Switzerland, including practitioners, networks of health care centers, professional associations, and health insurance companies, largely recognize that QCs are effective but they have difficulty generalizing the results with confidence. They believe that QCs provide a social context for reflective practice and allow the dissemination of knowledge to influence the work practices of the participants. While stakeholders agree about the context and broad range of internal mechanisms, they are not confident in understanding how the active components of QCs prompt physicians to change their behavior [31].

QCs have all the properties of complex interventions. Complex interventions depend on human behavior and their active ingredients tend to enable people to do the right thing at the right time or constrain them from doing something. They combine numerous and varying components and they function in diverse contexts. Individually, QCs respond to the unique constellation of the local needs of the complex system of primary health care. They are also responsive to changes in prevailing economic and cultural circumstances [32,33].

For these reasons, a realist review is necessary to explore how local context interacts with various mechanisms to produce more or less effective QCs [34-36]. Further, a realist review can produce important information about the relative effectiveness of various components, thereby enabling stakeholders and practitioners to make informed decisions about the best structure and process for their particular QCs.

### Objectives and focus of the review

The primary aim of this synthesis is to explain the QC program by finding underlying program theories, reviewing how the theories are tested within studies, and comparing context-mechanism-outcome (CMO) configurations across studies to produce a middle-range theory explaining why, how, and when QCs work, over what time frame, and in what circumstances. The range of components that characterize QCs, their underlying mechanisms, and the local context in which QCs are conducted will be documented. The patterns within QCs, in which components act both independently and inter-dependently, will be investigated and mapped in relation to variations in underlying mechanisms and the local context. Study outcomes will be evaluated to identify optimal conditions for success, which will then inform stakeholders about strategies to manage and maintain current QCs.

### Review questions

1) How do configurations of components and their underlying mechanisms within QCs influence their outcomes?

2) How do contextual features surrounding QCs improve individual and/or group performance?

## Methods

This protocol sets out the scope for the realist review, describes the initial program theory for QCs and relevant candidate theories, outlines the search strategy and process for sifting abstracts on the topic of interest, and proposes a theory-based framework for extracting data. Overall approaches to data analysis and synthesis are outlined, with the condition that, as with all realist reviews, the approaches are tailored to ensure they are appropriate for the available data.

### Identifying candidate theories

We initially explored the QC program theory using a scoping review of published papers and grey literature together with discussions with stakeholders [2,37-40]. By using terms such as ‘quality circle’ in the title and the abstract in Ovid MEDLINE and Embase, examining training material used in Switzerland, Austria and Germany, and studying the background literature used by stakeholders, the scoping review revealed a number of key elements that may facilitate QCs [4,5,41-44].

QCs are assumed to work because they bring people together to identify key issues concerning the quality of health care and they involve people in exploring solutions where there is a need for improvement. Coming from similar backgrounds, meeting in small groups at regular intervals, and, perhaps, voluntary participation are basic properties of QCs. It also seems to be important to have a trained facilitator who can engage the individuals and groups, support autonomy in terms of selection of the issue and the approach for reviewing information, and find solutions [3]. Communication techniques such as debate, consensus discussion, brainstorming, reflective thinking, self-observation, and role play among other practices appear to keep QCs active. Educational strategies such as audit and feedback, outreach visits, workshop-like atmosphere, and use of local opinion leaders are regularly brought into play [45,46]. Results of QCs include improved patient outcomes measured in changes of performance indicators and cost benefits [47].

After informal discussions with stakeholders from Switzerland and other European countries where QCs are prevalent, a set of questions was used to guide interviews with QC participants, facilitators, mentors, and regulators in Switzerland. These interviews clarified the objectives and the focus of QCs and confirmed that stakeholders have a common understanding of this important program. Stakeholders believe that QCs work because groups provide a social context for reflective practice and allow the dissemination of the findings to the participants’ working places, especially if they are embedded in a wider system. This has provided the impetus for this review.

### Search for data

An initial scoping search was performed to clarify the historical development of QCs, establish time frames, and describe their geographical distribution. The terms ‘peer review group’ (PRG), ‘quality circle’ (QC), and ‘small group work’ (SGW) were all included in the search as these terms are used interchangeably in different European countries. A Web of Knowledge citation map was used to identify the earliest published paper in 1979. Therefore, this search begins in 1980 using MEDLINE, Embase, PsycINFO, and CINHAL databases without language restrictions [48-51]. A comprehensive but purposive search for literature will be conducted using search strings for terms related to the descriptors of QC program theory, quality improvement, group functions and facilitation, and primary health care [Additional file 1]. In collaboration with a librarian, we developed a strategy that was guided by a selected set of documents [Additional file 2] [52]. At all stages, snowball strategies and access to the grey literature will be used. Authors will be contacted to clarify data when necessary. An iterative search may be necessary if new prospective theories are identified during data analysis. Results of stakeholder discussions, interviews, and consultations as well as training and conference material will serve as data.

### Selection of studies

Criteria for identifying relevant studies and appraising study designs have been piloted using a subset of relevant articles about the QCs identified during the scoping search. The two sifting questions for identifying relevant studies and papers will be:

1) Does the study describe QCs that take place in a primary health care setting?

2) Does the study describe QCs that include structured SGW including a facilitator?

These questions identify appropriate examples of QCs in the setting of primary health care. When both questions can be answered in the affirmative, the next step will be to identify appropriate quantitative papers that provide an adequate description of the evaluation methods and outcomes together with appropriate qualitative studies that provide descriptions and explanations of the key elements in the preliminary program theory.

Appropriate studies will be selected by asking:

1) Does the study provide details about the tools and outcomes of evaluation? Or

2) Does the study provide qualitative data on the context in which the QC takes place? Or

3) Does the study present qualitative data on QC group dynamics or facilitation? Or

4) Does the study contain qualitative data on QCs and social learning, adult learning, learning techniques, or behavioral change theories?

Quality assessment of the studies will focus on determining relevance and credibility in relation to the research question and how the findings relate to the context of the study [34]. Reasons why papers are included or excluded will be documented.

### Data extraction and analysis

Data will be extracted as text and quantitative summaries to describe the configuration of mechanisms, context, and outcomes for each study. Author discussions of reasons for QC successes or failures will be included as data. Where studies have explicitly used a program theory, the theory will be noted.

In the first stage of analysis, context components will be extracted together with descriptions of mechanisms on a study-by-study basis. The configuration within each study will be identified; that is, how context interacts with mechanisms to either enable or constrain QCs. Propositions will be developed for each study describing the relationship between context, mechanism, and outcome [53].

In the second stage of analysis, studies will be grouped by similar propositional statements in order to identify patterns in an iterative process. For example, QCs that occur in similar contexts could be compared to see if these similar contexts consistently trigger the same mechanisms. Likewise, QCs that focus on the same mechanisms will also be compared across different contexts to examine if these mechanisms are consistent in terms of producing similar outcomes. Differences in contexts and configurations of components will be highlighted in which the underlying mechanisms can either be constraining or enabling. These demi-regularities will be presented as statements of which types of QC work, in which local contexts and circumstances, for which type of practitioner, and at which points in time. Swiss and European stakeholders have declared an interest in assisting with building these propositional statements as experts in local contexts. At the third stage of analysis, middle-range theories will be explored in order to explain the visible and hidden forces behind the demi-regularities. Our consultation with stakeholders and reviews of training material suggested that we may find explanatory theories from psychological, social, or economic sciences that offer some explanation about the complex social interactions within a QC. The search on theories has revealed three different theoretical fields focusing on factors that influence professional behavior change: 1) theories of adult learning, social learning, and problem-based learning, which can be used to explain improvement in observed practice and changes in physician behavior [54,55]; 2) theories on individual practitioner behavior change, which can be mapped to general domains of behavior change [56]; and 3) theories related to implementation research in health settings and translating knowledge into action [57,58].

Over the past ten years, the theoretical domains that influence practitioner behavior change have been systematically identified [59,60]. The Theoretical Domains Framework (TDF) was developed as a theory-based classification of terms labeling behavior change across 14 key explanatory domains [61]. Several SRs and randomized studies have demonstrated that using theoretical domains helps to identify relevant theories to explain clinical practice and problems with implementation [62-64]. For this review, the TDF will be used as an initial organizing framework for categorizing the process of behavior change across QCs. It is recognized that most realist reviews aim to identify potential middle-range theories at an early stage of the review, but the scoping revealed a diversity in terms of types of theories. Therefore, we have chosen the approach used by Jagosh *et al*. (2013) of first sorting the literature and then immersion in the data, at which stage a considered approach can be taken to reviewing goodness of fit with the theoretical areas identified during preliminary theory scoping [65].

**Figure 1:**
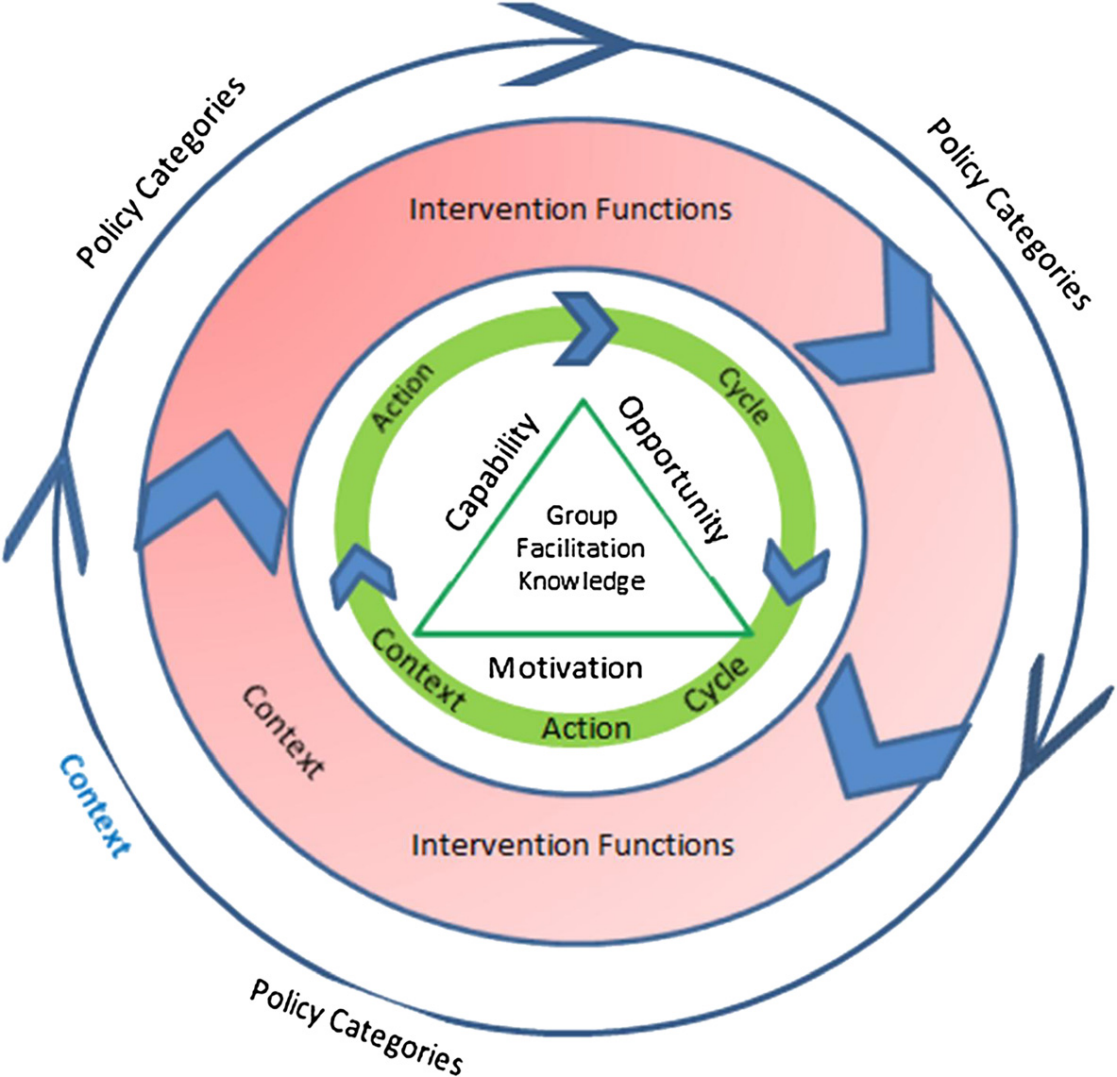
**Theoretical framework.** Center: group, group facilitation, and type of knowledge to be put into practice, representing the action cycle engine (PARiHS). Green triangle: interaction between motivation, opportunity, and capability, resulting in appropriate behavior for change (BCW). Green circle: knowledge-to-action cycle (KTAC). Red circle: intervention functions (BCW). Dark blue circle: policy categories (BCW). Blue circle: context factors on several levels of the program. BCW, behavior change wheel; KTAC, knowledge-to-action cycle; PARiHS, Promoting Action on Research Implementation in Health Services.

We will explore whether the configurations produced from our QC data can be mapped to three recently published theoretical frameworks: Promoting Action on Research Implementation in Health Services (PARiHS) framework, behavior change wheel (BCW), and knowledge-to-action cycle (KTAC) [56,66,67]. The PARiHS framework suggests that successful implementation requires an analysis of the nature and type of knowledge and the qualities of the local context. This analysis determines the type of facilitation that is needed to make the change successful. The BCW targets the necessary interacting conditions for individuals in a QC to start acting, by focusing on their capability, opportunity, and motivation. These conditions will boost the KTAC by facilitating individuals within the group to increase their capability and motivation after establishing favorable preconditions within QCs, depending on the type of knowledge gap. Using these three theoretical frameworks, a conceptual framework was developed for this review to allow the comparison of different theories, in a structure that is independent of their origin. This will be used for describing the core components of QCs, mapping the way in which they relate to each other, and for seeking possible reasons for these relationships (Figure 1).

### Data synthesis

We aim to synthesize the findings so that they are of use to practitioners when designing or modifying QCs. The stakeholders’ main interest in a realist review lies in learning more about which ingredients lead to better outcomes and lower costs and which contexts are necessary to achieve these goals over a longer period of time. Fine-tuning the synthesis of theories underlying each of the original review questions should provide stakeholders with relevant answers [34,53,68].

Theories in comparative settings will reveal which contextual features are of importance. Questioning program integrity will reveal which combinations of contexts or interventions at different levels, be it group, organization, or primary health care, will support the QC process. The assessment of rival program theories will identify middle-range theories and demi-regularities that can optimize QC performance. This will result in specific recommendations of optimal variations to the program in specific contexts, thus enabling stakeholders to implement successful patterns of contextual features and effective QC components [53].

### Reporting and dissemination of the findings

Transparent reporting of the process and findings is important for creating an audit trail. The standards of realist and meta-narrative evidence synthesis (RAMESES) provide publication criteria for a review of this type [69]. The authors will follow the RAMESES statement as they explore and answer the research questions in language acceptable to all stakeholders. An academic article will be written for publication in an international journal specializing in implementation. Findings will be disseminated through consultations with stakeholders in Switzerland, who will be able to critically evaluate the results and then, ultimately, implement them. Further, the results of the review will be presented at the meeting of the European Society for Quality and Safety in Family Practice (EQuiP) in 2015. This will allow European stakeholders to evaluate current practice and discuss further steps for enhancing QCs across Europe.

### Trial status

The authors have already completed the protocol, and plan in December 2013 to start the review, which has been registered with PROSPERO, the international prospective register of systematic reviews: CRD42013004826.

## Discussion

### Ethical issues

A review of this kind does not require approval from an ethics committee because it is not primary research. However, it will follow the relevant standards of utility, usefulness, feasibility, propriety, accuracy, and accountability [70,71].

### Limitations

This realist review has two major limitations. Firstly, it is dependent on the transparency and adequacy of reporting by original authors. To address this, authors will be contacted for clarification and related reports will be examined. Additionally, there is the risk of the selective bias of choosing underlying theories and synthesizing them in an *ad hoc* manner. This will be addressed through the creation of an overarching framework to guide systematic theory development and through an iterative process of communication with the stakeholders and consultation of the literature.

### Summary

Realist synthesis is a methodology under development and this protocol makes explicit the processes of using stakeholders as a key source of information to clarify the scope of the review. We developed separate search strategies for understanding the scope, finding underlying theories, and identifying primary studies. We explicitly described the data extraction and analysis process across three levels of investigation, and have planned the final stage of synthesizing data and drawing of conclusions for theory development. The stakeholders will receive on-going reports for checking and ensuring policy relevance and for safeguarding future policy development.

## Abbreviations

BCW: Behavior change wheel; CME: Continuing medical education; CMO: Context-mechanism-outcome; CPD: Continuing professional development; EQuiP: European Society for Quality and Safety in Family Practice; KTAC: Knowledge-to-action cycle; PARiHS: Promoting action on research implementation in health services; PRG: Peer review group; QC: Quality circle; RAMESES: Realist and meta-narrative evidence synthesis; SGW: Small group work; SR: Systematic review; TDF: Theoretical domains framework.

## Competing interests

There are no financial conflicts. As a member of the committee of quality in Swiss family practices and a supervisor and trainer of facilitators working at santémed Health Care Centres, a network providing primary health care services, AR has great personal expertise and many years’ experience of QCs. SM and JH contribute independently to this project from their academic and methodological experience.

## Authors’ contributions

AR performed the conceptual work and prepared the protocol as part of formal postgraduate study (DPhil Programme in Evidence-Based Health Care, University of Oxford, Oxford, UK). SM supervised the development of the protocol, critically reviewed the text, and assisted with editing. JH provided important input regarding the methodology and revised the protocol with regard to the realist approach. All authors read and approved the final manuscript.

## Authors’ information

AR: DPhil, Department of Primary Health Care Sciences, University of Oxford, Oxford, UK; santémed Health Care Centres; member of EQuiP; and delegate for Quality in Medicine on behalf of the Swiss Association of Family Medicine. SM: Senior Researcher, Centre for Evidence Based Medicine, Department of Primary Health Care Sciences, University of Oxford, Oxford, UK. JH: Senior Lecturer and Course Director, MPH in International Health Management and Leadership, School of Health and Related Research (ScHARR), University of Sheffield, Sheffield, UK; and Co-Convenor, Cochrane Qualitative Research Methods Group.

## Supplementary Material

Additional file 1 Program’, ‘quality improvement’, ‘group’, and ‘primary care’ terms. Click here for file

Additional file 2 Search strategy in Ovid MEDLINE, Embase, and PsycINFO. Click here for file
