# Supplementary material for: Exploring why quality circles work in primary health care: a realist review protocol
Source: Syst Rev. 2013 Dec 9;2:110. doi: 10.1186/2046-4053-2-110 (PMC4029275; doi:10.1186/2046-4053-2-110)
Supplement: Additional file 1 — Program’, ‘quality improvement’, ‘group’, and ‘primary care’ terms. [file 2046-4053-2-110-S1.docx]

**“Programme” terms**

|  | Programme terms |
| --- | --- |
| 1 | Management Quality Circles/ |
| 2 | Quality circle*.ti,ab. |
| 3 | (Group* adj3 (learning or work* or teaching or education*)).ti,ab |
| 4 | (Group* adj2 (intervention* or strateg* or program* or review*)).ti,ab. |
| 5 | (Quality improvement* adj3 (intervention* or strateg* or program* or initiative* or tool*)).ti,ab. |
| 6 | (Audit adj3 feedback).ti,ab. |
| 7 | Peer review*.ti,ab. |
| 8 | Reflective practice.ti,ab. |
| 9 | (Learning adj3 (intervention* or strateg* or program* or initiative*)).ti,ab. |
| 10 | (Education* adj3 (intervention* or strateg* or program* or initiative*)).ti,ab. |
| 11 | (Continuing adj2 (education or development)).ti,ab. |
| 12 | Peer Review, Health Care/ |
| 13 | Medical audit/ or nursing audit/ |
| 14 | exp Education, Continuing/ |
| 15 | 1 or 2 or 3 or 4 or 5 or 6 or 7 or 8 or 9 or 10 or 11 or 12 or 13 or 14 |

**“Quality Improvement” terms**

|  | Quality Improvement terms |  |
| --- | --- | --- |
| 1 | Quality Assurance, Health Care/Total Quality Management/ |  |
| 2 | Quality Improvement/ |  |
| 3 | "Quality of Health Care"/ |  |
| 4 | Evidence-based practice/ or evidence-based medicine/ or evidence-based nursing/ |  |
| 5 | Physician's Practice Patterns/ |  |
| 6 | exp Professional Competence/ |  |
| 7 | Guideline Adherence/ |  |
| 8 | (Quality adj3 (improv* or assurance or change)).ti,ab. |  |
| 9 | (Practice adj3 (improv* or change)).ti,ab. |  |
| 10 | ((Care or healthcare) adj3 (improv* or change)).ti,ab. |  |
| 11 | ((Professional or physician* or medical or clinical or nurs*) adj competenc*).ti,ab. |  |
| 12 | ((Guideline* or guidance or standard* or protocol*) adj2 (adhere* or complian* or concord* or implement*)).ti,ab. |  |
| 13 | (Evidence based adj2 (practice or prescrib*)).ti,ab. |  |
| 14 | 1 or 2 or 3 or 4 or 5 or 6 or 7 or 8 or 9 or 10 or 11 or 12 or 13 |  |
|  |  |  |

**“Group” terms**

|  | Group terms |
| --- | --- |
| 1 | Peer Groups/ |
| 2 | Group*.ti,ab |
| 3 | Group Process/ |
| 4 | Group Practice/ |
| 5  6  7  8 | Practice based.ti,ab  Facilitation.ti,ab  Facilitator.ti,ab  1 or 2 or 3 or 4 or 5 or 6 or 7 |

**“Primary Care” terms:**

|  | Primary Care Terms |  |
| --- | --- | --- |
| 1 | General practice/ or family practice/ |  |
| 2 | Primary Health Care/ |  |
| 3 | General practitioners/ or physicians, family/ or physicians, primary care/ |  |
| 4 | Community health services/ or community health nursing/ or community mental health services/ |  |
| 5 | (Family adj3 (practice or practitioner* or physician*)).ti,ab. |  |
| 6 | (General adj3 (practice or practitioner* or physician*)).ti,ab. |  |
| 7 | (Primary adj3 (care or healthcare)).ti,ab. |  |
| 8 | (Community adj2 nurs*).ti,ab. |  |
| 9 | 1 or 2 or 3 or 4 or 5 or 6 or 7 or 8 |  |
