# Supplementary material for: Exploring why quality circles work in primary health care: a realist review protocol
Source: Syst Rev. 2013 Dec 9;2:110. doi: 10.1186/2046-4053-2-110 (PMC4029275; doi:10.1186/2046-4053-2-110)
Supplement: Additional file 2 — Search strategy in Ovid MEDLINE, Embase, and PsycINFO. [file 2046-4053-2-110-S2.docx]

**Search Strategy in OVID Medline / EMBASE / PsycInfo**

1. general practice/ or family practice/

2. Primary Health Care/

3. general practitioners/ or physicians, family/ or physicians, primary care/

4. community health services/ or community health nursing/ or community mental health services/

5. (family adj3 (practice or practitioner* or physician*)).ti,ab.

6. (general adj3 (practice or practitioner* or physician*)).ti,ab.

7. (primary adj3 (care or healthcare)).ti,ab.

8. practice nurs*.ti,ab.

9. (community adj2 nurs*).ti,ab.

**PRIMARY HEALTH CARE TERMS**

10. 1 or 2 or 3 or 4 or 5 or 6 or 7 or 8 or 9

11. Management Quality Circles/

12. quality circle*.ti,ab.

13. (group* adj3 (learning or work* or teaching or education*)).ti,ab.

14. (group* adj2 (intervention* or strateg* or program* or review*)).ti,ab.

15. (quality improvement* adj3 (intervention* or strateg* or program* or initiative* or tool*)).ti,ab.

16. (audit adj3 feedback).ti,ab.

17. peer review*.ti,ab.

18. reflective practice.ti,ab.

19. (learning adj3 (intervention* or strateg* or program* or initiative*)).ti,ab.

20. (education* adj3 (intervention* or strateg* or program* or initiative*)).ti,ab.

21. (continuing adj2 (education or development)).ti,ab.

22. Peer Review, Health Care/

23. medical audit/ or nursing audit/

24. exp Education, Continuing/

**PROGRAMME TERMS**

25. 11 or 12 or 13 or 14 or 15 or 16 or 17 or 18 or 19 or 20 or 21 or 22 or 23 or 24

26. Quality Assurance, Health Care/

27. Total Quality Management/

28. Quality Improvement/

29. "Quality of Health Care"/

30. evidence-based practice/ or evidence-based medicine/ or evidence-based nursing/

31. Physician's Practice Patterns/

32. exp Professional Competence/

33. Guideline Adherence/

34. (quality adj3 (improv* or assurance or change)).ti,ab.

35. (practice adj3 (improv* or change)).ti,ab.

36. ((care or healthcare) adj3 (improv* or change)).ti,ab.

37. ((professional or physician* or medical or clinical or nurs*) adj competenc*).ti,ab.

38. ((guideline* or guidance or standard* or protocol*) adj2 (adhere* or complian* or concord* or implement*)).ti,ab.

39. (evidence based adj2 (practice or prescrib*)).ti,ab.

**TERMS for QUALITY IMPROVEMENT**

40. 26 or 27 or 28 or 29 or 30 or 31 or 32 or 33 or 34 or 35 or 36 or 37 or 38 or 39

41. Peer Groups/

42. Group Process/

43. Group Practice/

44. practice based.ti,ab.

**GROUP TERMS**

45. 41 or 42 or 43 or 44

**ADDITIONAL GROUP TERM**

46. facilitator.ti,ab.

GROUP TERMS IN PRIMARY CARE

47. 10 and 46

PRIMARY CARE **AND** PROGRAM TERMS **AND**  QUALITY IMPROVEMENT TERMS **AND** GROUP TERMS

48. 10 and 25 and 40 and 45

ADDING THE “QUALITY CIRCLES” **AND** “GROUP FACILITATION” IN TITEL **AND** ABSTRACT

49. 12 or 47 or 48

50. limit 49 to yr="1980 -Current"

**Search Strategy in EBSCO CINAHL**

| # | Query | Limiters/Expanders | Last Run Via | Results | Action |
| --- | --- | --- | --- | --- | --- |
| S62 | S17 OR S18 OR S61 | Search modes - Boolean/Phrase | Interface - EBSCOhost Research Databases  Search Screen - Advanced Search  Database - CINAHL | 530 | [Edit](javascript:__doPostBack('ctl00$ctl00$MainContentArea$MainContentArea$editControl$printHistory$HistoryRepeater$ctl00$linkEditSearch','')) S62 |
| S61 | S58 AND S59 AND S60 | Search modes - Boolean/Phrase | Interface - EBSCOhost Research Databases  Search Screen - Advanced Search  Database - CINAHL | 317 | [Edit](javascript:__doPostBack('ctl00$ctl00$MainContentArea$MainContentArea$editControl$printHistory$HistoryRepeater$ctl01$linkEditSearch','')) S61 |
| S60 | S53 OR S54 OR S55 OR S56 OR S57 | Search modes - Boolean/Phrase  **GROUP TERMS** | Interface - EBSCOhost Research Databases  Search Screen - Advanced Search  Database - CINAHL | 11,357 | [Edit](javascript:__doPostBack('ctl00$ctl00$MainContentArea$MainContentArea$editControl$printHistory$HistoryRepeater$ctl02$linkEditSearch','')) S60 |
| S59 | S35 OR S36 OR S37 OR S38 OR S39 OR S40 OR S41 OR S42 OR S43 OR S44 OR S45 OR S46 OR S47 OR S48 OR S49 OR S50 OR S51 OR S52 | Search modes - Boolean/Phrase  **TERMS for QUALITY IMPROVEMENT** | Interface - EBSCOhost Research Databases  Search Screen - Advanced Search  Database - CINAHL | 161,756 | [Edit](javascript:__doPostBack('ctl00$ctl00$MainContentArea$MainContentArea$editControl$printHistory$HistoryRepeater$ctl03$linkEditSearch','')) S59 |
| S58 | S17 OR S18 OR S19 OR S20 OR S21 OR S22 OR S23 OR S24 OR S25 OR S26 OR S27 OR S28 OR S29 OR S30 OR S31 OR S32 OR S33 OR S34 | Search modes - Boolean/Phrase  **PROGRAMME TERMS** | Interface - EBSCOhost Research Databases  Search Screen - Advanced Search  Database - CINAHL | 182,211 | [Edit](javascript:__doPostBack('ctl00$ctl00$MainContentArea$MainContentArea$editControl$printHistory$HistoryRepeater$ctl04$linkEditSearch','')) S58 |
| S57 | TI facilitator OR AB facilitator | Search modes - Boolean/Phrase | Interface - EBSCOhost Research Databases  Search Screen - Advanced Search  Database - CINAHL | 1,087 | [Edit](javascript:__doPostBack('ctl00$ctl00$MainContentArea$MainContentArea$editControl$printHistory$HistoryRepeater$ctl05$linkEditSearch','')) S57 |
| S56 | (MH "Learning Facilitation (Iowa NIC)") OR "facilitator" | Search modes - Boolean/Phrase | Interface - EBSCOhost Research Databases  Search Screen - Advanced Search  Database - CINAHL | 1,088 | [Edit](javascript:__doPostBack('ctl00$ctl00$MainContentArea$MainContentArea$editControl$printHistory$HistoryRepeater$ctl06$linkEditSearch','')) S56 |
| S55 | (MH "Group Practice") OR "group practice" | Search modes - Boolean/Phrase | Interface - EBSCOhost Research Databases  Search Screen - Advanced Search  Database - CINAHL | 1,525 | [Edit](javascript:__doPostBack('ctl00$ctl00$MainContentArea$MainContentArea$editControl$printHistory$HistoryRepeater$ctl07$linkEditSearch','')) S55 |
| S54 | (MH "Group Processes") OR "group process" | Search modes - Boolean/Phrase | Interface - EBSCOhost Research Databases  Search Screen - Advanced Search  Database - CINAHL | 4,527 | [Edit](javascript:__doPostBack('ctl00$ctl00$MainContentArea$MainContentArea$editControl$printHistory$HistoryRepeater$ctl08$linkEditSearch','')) S54 |
| S53 | (MH "Peer Group") OR (MH "Group Processes") OR "peer groups" | Search modes - Boolean/Phrase | Interface - EBSCOhost Research Databases  Search Screen - Advanced Search  Database - CINAHL | 8,583 | [Edit](javascript:__doPostBack('ctl00$ctl00$MainContentArea$MainContentArea$editControl$printHistory$HistoryRepeater$ctl09$linkEditSearch','')) S53 |
| S52 | TI ( (evidence based n2 (practice or prescrib*)) ) OR AB ( (evidence based n2 (practice or prescrib*)) ) | Search modes - Boolean/Phrase | Interface - EBSCOhost Research Databases  Search Screen - Advanced Search  Database - CINAHL | 7,657 | [Edit](javascript:__doPostBack('ctl00$ctl00$MainContentArea$MainContentArea$editControl$printHistory$HistoryRepeater$ctl10$linkEditSearch','')) S52 |
| S51 | TI ( ((guideline* or guidance or standard* or protocol*) n2 (adhere* or complian* or concord* or implement*)). ) OR AB ( ((guideline* or guidance or standard* or protocol*) n2 (adhere* or complian* or concord* or implement*)). ) | Search modes - Boolean/Phrase | Interface - EBSCOhost Research Databases  Search Screen - Advanced Search  Database - CINAHL | 5,175 | [Edit](javascript:__doPostBack('ctl00$ctl00$MainContentArea$MainContentArea$editControl$printHistory$HistoryRepeater$ctl11$linkEditSearch','')) S51 |
| S50 | (MH "Professional Competence") OR "clinical compet*" | Search modes - Boolean/Phrase | Interface - EBSCOhost Research Databases  Search Screen - Advanced Search  Database - CINAHL | 25,241 | [Edit](javascript:__doPostBack('ctl00$ctl00$MainContentArea$MainContentArea$editControl$printHistory$HistoryRepeater$ctl12$linkEditSearch','')) S50 |
| S49 | "medical competenc*" OR (MH "Medical Practice") | Search modes - Boolean/Phrase | Interface - EBSCOhost Research Databases  Search Screen - Advanced Search  Database - CINAHL | 3,397 | [Edit](javascript:__doPostBack('ctl00$ctl00$MainContentArea$MainContentArea$editControl$printHistory$HistoryRepeater$ctl13$linkEditSearch','')) S49 |
| S48 | "physician* competence" OR (MH "Competency Assessment") | Search modes - Boolean/Phrase | Interface - EBSCOhost Research Databases  Search Screen - Advanced Search  Database - CINAHL | 2,658 | [Edit](javascript:__doPostBack('ctl00$ctl00$MainContentArea$MainContentArea$editControl$printHistory$HistoryRepeater$ctl14$linkEditSearch','')) S48 |
| S47 | (MH "Professional Competence") OR (MH "Clinical Competence") OR "professional competenc*" | Search modes - Boolean/Phrase | Interface - EBSCOhost Research Databases  Search Screen - Advanced Search  Database - CINAHL | 25,144 | [Edit](javascript:__doPostBack('ctl00$ctl00$MainContentArea$MainContentArea$editControl$printHistory$HistoryRepeater$ctl15$linkEditSearch','')) S47 |
| S46 | TI ( ((care or healthcare) n3 (improv* or change)) ) OR AB ( ((care or healthcare) n3 (improv* or change)) ) | Search modes - Boolean/Phrase | Interface - EBSCOhost Research Databases  Search Screen - Advanced Search  Database - CINAHL | 22,188 | [Edit](javascript:__doPostBack('ctl00$ctl00$MainContentArea$MainContentArea$editControl$printHistory$HistoryRepeater$ctl16$linkEditSearch','')) S46 |
| S45 | TI ( (practice n3 (improv* or change)) ) OR AB ( (practice n3 (improv* or change)) ) | Search modes - Boolean/Phrase | Interface - EBSCOhost Research Databases  Search Screen - Advanced Search  Database - CINAHL | 6,255 | [Edit](javascript:__doPostBack('ctl00$ctl00$MainContentArea$MainContentArea$editControl$printHistory$HistoryRepeater$ctl17$linkEditSearch','')) S45 |
| S44 | TI ( (quality n3 (improv* or assurance or change)) ) OR AB ( (quality n3 (improv* or assurance or change)) ) | Search modes - Boolean/Phrase | Interface - EBSCOhost Research Databases  Search Screen - Advanced Search  Database - CINAHL | 25,900 | [Edit](javascript:__doPostBack('ctl00$ctl00$MainContentArea$MainContentArea$editControl$printHistory$HistoryRepeater$ctl18$linkEditSearch','')) S44 |
| S43 | (MH "Guideline Adherence") OR "Guideline Adherence" | Search modes - Boolean/Phrase | Interface - EBSCOhost Research Databases  Search Screen - Advanced Search  Database - CINAHL | 3,289 | [Edit](javascript:__doPostBack('ctl00$ctl00$MainContentArea$MainContentArea$editControl$printHistory$HistoryRepeater$ctl19$linkEditSearch','')) S43 |
| S42 | (MH "Professional Competence") OR "exp Professional Competence" OR (MH "Clinical Competence") OR (MH "Competency Assessment") | Search modes - Boolean/Phrase | Interface - EBSCOhost Research Databases  Search Screen - Advanced Search  Database - CINAHL | 26,533 | [Edit](javascript:__doPostBack('ctl00$ctl00$MainContentArea$MainContentArea$editControl$printHistory$HistoryRepeater$ctl20$linkEditSearch','')) S42 |
| S41 | (MH "Practice Patterns") OR "Physician's Practice Patterns" | Search modes - Boolean/Phrase | Interface - EBSCOhost Research Databases  Search Screen - Advanced Search  Database - CINAHL | 4,201 | [Edit](javascript:__doPostBack('ctl00$ctl00$MainContentArea$MainContentArea$editControl$printHistory$HistoryRepeater$ctl21$linkEditSearch','')) S41 |
| S40 | (MH "Nursing Practice, Evidence-Based") OR (MH "Physical Therapy Practice, Evidence-Based") OR (MH "Occupational Therapy Practice, Evidence-Based") OR (MH "Evidence-Based Dental Practice") OR (MH "Medical Practice, Evidence-Based") OR (MH "Professional Practice, Evidence-Based") OR (MH "Education, Nursing, Research-Based") OR (MH "Education, Nursing, Theory-Based") OR (MH "Nursing Practice, Research-Based") OR "evidence-based nursing" | Search modes - Boolean/Phrase | Interface - EBSCOhost Research Databases  Search Screen - Advanced Search  Database - CINAHL | 35,190 | [Edit](javascript:__doPostBack('ctl00$ctl00$MainContentArea$MainContentArea$editControl$printHistory$HistoryRepeater$ctl22$linkEditSearch','')) S40 |
| S39 | (MH "Medical Practice, Evidence-Based") OR (MH "Physical Therapy Practice, Evidence-Based") OR (MH "Occupational Therapy Practice, Evidence-Based") OR (MH "Evidence-Based Dental Practice") OR (MH "Nursing Practice, Evidence-Based") OR (MH "Professional Practice, Evidence-Based") OR (MH "Medical Practice, Research-Based") OR "evidence-based medicine" | Search modes - Boolean/Phrase | Interface - EBSCOhost Research Databases  Search Screen - Advanced Search  Database - CINAHL | 34,469 | [Edit](javascript:__doPostBack('ctl00$ctl00$MainContentArea$MainContentArea$editControl$printHistory$HistoryRepeater$ctl23$linkEditSearch','')) S39 |
| S38 | (MH "Quality of Health Care") OR "Quality of Health Care" | Search modes - Boolean/Phrase | Interface - EBSCOhost Research Databases  Search Screen - Advanced Search  Database - CINAHL | 35,603 | [Edit](javascript:__doPostBack('ctl00$ctl00$MainContentArea$MainContentArea$editControl$printHistory$HistoryRepeater$ctl24$linkEditSearch','')) S38 |
| S37 | (MH "Quality Improvement") OR "Quality Improvement" | Search modes - Boolean/Phrase | Interface - EBSCOhost Research Databases  Search Screen - Advanced Search  Database - CINAHL | 24,125 | [Edit](javascript:__doPostBack('ctl00$ctl00$MainContentArea$MainContentArea$editControl$printHistory$HistoryRepeater$ctl25$linkEditSearch','')) S37 |
| S36 | (MH "Quality Improvement") OR (MH "Quality Management, Organizational") OR "Total Quality Management" | Search modes - Boolean/Phrase | Interface - EBSCOhost Research Databases  Search Screen - Advanced Search  Database - CINAHL | 22,025 | [Edit](javascript:__doPostBack('ctl00$ctl00$MainContentArea$MainContentArea$editControl$printHistory$HistoryRepeater$ctl26$linkEditSearch','')) S36 |
| S35 | (MH "Quality of Care Research") OR (MH "Quality of Health Care") OR "Quality Assurance, Health Care" | Search modes - Boolean/Phrase | Interface - EBSCOhost Research Databases  Search Screen - Advanced Search  Database - CINAHL | 35,757 | [Edit](javascript:__doPostBack('ctl00$ctl00$MainContentArea$MainContentArea$editControl$printHistory$HistoryRepeater$ctl27$linkEditSearch','')) S35 |
| S34 | (MH "Education, Medical, Continuing") OR (MH "Education, Nursing, Continuing") OR (MH "Education, Continuing") OR (MH "Education, Continuing (Credit)") OR "exp Education, Continuing" | Search modes - Boolean/Phrase | Interface - EBSCOhost Research Databases  Search Screen - Advanced Search  Database - CINAHL | 90,236 | [Edit](javascript:__doPostBack('ctl00$ctl00$MainContentArea$MainContentArea$editControl$printHistory$HistoryRepeater$ctl28$linkEditSearch','')) S34 |
| S33 | (MH "Nursing Audit") OR "nursing audit" | Search modes - Boolean/Phrase | Interface - EBSCOhost Research Databases  Search Screen - Advanced Search  Database - CINAHL | 647 | [Edit](javascript:__doPostBack('ctl00$ctl00$MainContentArea$MainContentArea$editControl$printHistory$HistoryRepeater$ctl29$linkEditSearch','')) S33 |
| S32 | "medical audit" OR (MH "Record Review") OR (MH "Nursing Audit") OR (MH "Audit") OR (MH "Medical Practice") OR (MH "Medical Transcription") OR (MH "Health Care Errors") | Search modes - Boolean/Phrase | Interface - EBSCOhost Research Databases  Search Screen - Advanced Search  Database - CINAHL | 47,994 | [Edit](javascript:__doPostBack('ctl00$ctl00$MainContentArea$MainContentArea$editControl$printHistory$HistoryRepeater$ctl30$linkEditSearch','')) S32 |
| S31 | TI ( (continuing n2 (education or development)) ) OR AB ( (continuing n2 (education or development)) ) | Search modes - Boolean/Phrase | Interface - EBSCOhost Research Databases  Search Screen - Advanced Search  Database - CINAHL | 10,631 | [Edit](javascript:__doPostBack('ctl00$ctl00$MainContentArea$MainContentArea$editControl$printHistory$HistoryRepeater$ctl31$linkEditSearch','')) S31 |
| S30 | TI ( (education* n3 (intervention* or strateg* or program* or initiative*)) ) OR AB ( (education* n3 (intervention* or strateg* or program* or initiative*)) ) | Search modes - Boolean/Phrase | Interface - EBSCOhost Research Databases  Search Screen - Advanced Search  Database - CINAHL | 24,242 | [Edit](javascript:__doPostBack('ctl00$ctl00$MainContentArea$MainContentArea$editControl$printHistory$HistoryRepeater$ctl32$linkEditSearch','')) S30 |
| S29 | TI ( (learning n3 (intervention* or strateg* or program* or initiative*)) ) OR AB ( (learning n3 (intervention* or strateg* or program* or initiative*)) ) | Search modes - Boolean/Phrase | Interface - EBSCOhost Research Databases  Search Screen - Advanced Search  Database - CINAHL | 3,317 | [Edit](javascript:__doPostBack('ctl00$ctl00$MainContentArea$MainContentArea$editControl$printHistory$HistoryRepeater$ctl33$linkEditSearch','')) S29 |
| S28 | TI reflective practice | Search modes - Boolean/Phrase | Interface - EBSCOhost Research Databases  Search Screen - Advanced Search  Database - CINAHL | 429 | [Edit](javascript:__doPostBack('ctl00$ctl00$MainContentArea$MainContentArea$editControl$printHistory$HistoryRepeater$ctl34$linkEditSearch','')) S28 |
| S27 | TI peer review* OR AB peer review* | Search modes - Boolean/Phrase | Interface - EBSCOhost Research Databases  Search Screen - Advanced Search  Database - CINAHL | 3,731 | [Edit](javascript:__doPostBack('ctl00$ctl00$MainContentArea$MainContentArea$editControl$printHistory$HistoryRepeater$ctl35$linkEditSearch','')) S27 |
| S26 | TI (audit n3 feedback) OR AB (audit n3 feedback) | Search modes - Boolean/Phrase | Interface - EBSCOhost Research Databases  Search Screen - Advanced Search  Database - CINAHL | 188 | [Edit](javascript:__doPostBack('ctl00$ctl00$MainContentArea$MainContentArea$editControl$printHistory$HistoryRepeater$ctl36$linkEditSearch','')) S26 |
| S25 | TI ( (quality improvement* n3 (intervention* or strateg* or program* or initiative* or tool*)) ) OR AB ( (quality improvement* n3 (intervention* or strateg* or program* or initiative* or tool*)) ) | Search modes - Boolean/Phrase | Interface - EBSCOhost Research Databases  Search Screen - Advanced Search  Database - CINAHL | 2,097 | [Edit](javascript:__doPostBack('ctl00$ctl00$MainContentArea$MainContentArea$editControl$printHistory$HistoryRepeater$ctl37$linkEditSearch','')) S25 |
| S24 | TI ( (group* n2 (intervention* or strateg* or program* or review*)) ) OR AB ( (group* n2 (intervention* or strateg* or program* or review*)) ) | Search modes - Boolean/Phrase | Interface - EBSCOhost Research Databases  Search Screen - Advanced Search  Database - CINAHL | 13,062 | [Edit](javascript:__doPostBack('ctl00$ctl00$MainContentArea$MainContentArea$editControl$printHistory$HistoryRepeater$ctl38$linkEditSearch','')) S24 |
| S23 | (MH "Teaching: Group (Iowa NIC)") OR (MH "Group Exercise") OR "group teaching" | Search modes - Boolean/Phrase | Interface - EBSCOhost Research Databases  Search Screen - Advanced Search  Database - CINAHL | 379 | [Edit](javascript:__doPostBack('ctl00$ctl00$MainContentArea$MainContentArea$editControl$printHistory$HistoryRepeater$ctl39$linkEditSearch','')) S23 |
| S22 | (MH "Group Exercise") OR "group education" | Search modes - Boolean/Phrase | Interface - EBSCOhost Research Databases  Search Screen - Advanced Search  Database - CINAHL | 633 | [Edit](javascript:__doPostBack('ctl00$ctl00$MainContentArea$MainContentArea$editControl$printHistory$HistoryRepeater$ctl40$linkEditSearch','')) S22 |
| S21 | (MH "Education, Social Work") OR "group work in education" | Search modes - Boolean/Phrase | Interface - EBSCOhost Research Databases  Search Screen - Advanced Search  Database - CINAHL | 1,998 | [Edit](javascript:__doPostBack('ctl00$ctl00$MainContentArea$MainContentArea$editControl$printHistory$HistoryRepeater$ctl41$linkEditSearch','')) S21 |
| S20 | (MH "Group Exercise") OR "group learning" | Search modes - Boolean/Phrase | Interface - EBSCOhost Research Databases  Search Screen - Advanced Search  Database - CINAHL | 444 | [Edit](javascript:__doPostBack('ctl00$ctl00$MainContentArea$MainContentArea$editControl$printHistory$HistoryRepeater$ctl42$linkEditSearch','')) S20 |
| S19 | "group work" | Search modes - Boolean/Phrase | Interface - EBSCOhost Research Databases  Search Screen - Advanced Search  Database - CINAHL | 709 | [Edit](javascript:__doPostBack('ctl00$ctl00$MainContentArea$MainContentArea$editControl$printHistory$HistoryRepeater$ctl43$linkEditSearch','')) S19 |
| S18 | TI quality circle* OR AB quality circle* | Search modes - Boolean/Phrase | Interface - EBSCOhost Research Databases  Search Screen - Advanced Search  Database - CINAHL | 134 | [Edit](javascript:__doPostBack('ctl00$ctl00$MainContentArea$MainContentArea$editControl$printHistory$HistoryRepeater$ctl44$linkEditSearch','')) S18 |
| S17 | (MH "Quality Circles") OR "Management Quality Circles" | Search modes - Boolean/Phrase | Interface - EBSCOhost Research Databases  Search Screen - Advanced Search  Database - CINAHL | 110 | [Edit](javascript:__doPostBack('ctl00$ctl00$MainContentArea$MainContentArea$editControl$printHistory$HistoryRepeater$ctl45$linkEditSearch','')) S17 |
| S16 | S1 OR S2 OR S3 OR S4 OR S5 OR S6 OR S7 OR S8 OR S9 OR S10 OR S11 OR S12 OR S13 OR S14 OR S15 | Search modes - Boolean/Phrase  **PRIMARY HEALTH CARE TERMS!** | Interface - EBSCOhost Research Databases  Search Screen - Advanced Search  Database - CINAHL | 131,279 | [Edit](javascript:__doPostBack('ctl00$ctl00$MainContentArea$MainContentArea$editControl$printHistory$HistoryRepeater$ctl46$linkEditSearch','')) S16 |
| S15 | TI (community n2 nurs*) OR AB (community n2 nurs*) | Search modes - Boolean/Phrase | Interface - EBSCOhost Research Databases  Search Screen - Advanced Search  Database - CINAHL | 7,234 | [Edit](javascript:__doPostBack('ctl00$ctl00$MainContentArea$MainContentArea$editControl$printHistory$HistoryRepeater$ctl47$linkEditSearch','')) S15 |
| S14 | TI practice nurs* OR AB practice nurs* | Search modes - Boolean/Phrase | Interface - EBSCOhost Research Databases  Search Screen - Advanced Search  Database - CINAHL | 35,755 | [Edit](javascript:__doPostBack('ctl00$ctl00$MainContentArea$MainContentArea$editControl$printHistory$HistoryRepeater$ctl48$linkEditSearch','')) S14 |
| S13 | TI ( (primary n3 (care or health care)) ) OR AB ( (primary n3 (care or health care)) ) | Search modes - Boolean/Phrase | Interface - EBSCOhost Research Databases  Search Screen - Advanced Search  Database - CINAHL | 32,317 | [Edit](javascript:__doPostBack('ctl00$ctl00$MainContentArea$MainContentArea$editControl$printHistory$HistoryRepeater$ctl49$linkEditSearch','')) S13 |
| S12 | TI ( (primary n3 (care or healthcare)) ) OR AB ( (primary n3 (care or healthcare)) ) | Search modes - Boolean/Phrase | Interface - EBSCOhost Research Databases  Search Screen - Advanced Search  Database - CINAHL | 32,964 | [Edit](javascript:__doPostBack('ctl00$ctl00$MainContentArea$MainContentArea$editControl$printHistory$HistoryRepeater$ctl50$linkEditSearch','')) S12 |
| S11 | TI ( (general n3 (practice or practitioner* or physician*)) ) OR AB ( (general n3 (practice or practitioner* or physician*)) ) | Search modes - Boolean/Phrase | Interface - EBSCOhost Research Databases  Search Screen - Advanced Search  Database - CINAHL | 11,146 | [Edit](javascript:__doPostBack('ctl00$ctl00$MainContentArea$MainContentArea$editControl$printHistory$HistoryRepeater$ctl51$linkEditSearch','')) S11 |
| S10 | TI ( (family n3 (practice or practitioner* or physician*)) ) OR AB ( (family n3 (practice or practitioner* or physician*)) ) | Search modes - Boolean/Phrase | Interface - EBSCOhost Research Databases  Search Screen - Advanced Search  Database - CINAHL | 5,270 | [Edit](javascript:__doPostBack('ctl00$ctl00$MainContentArea$MainContentArea$editControl$printHistory$HistoryRepeater$ctl52$linkEditSearch','')) S10 |
| S9 | (MH "Community Mental Health Services") OR "community mental health services" OR (MH "Community Mental Health Nursing") | Search modes - Boolean/Phrase | Interface - EBSCOhost Research Databases  Search Screen - Advanced Search  Database - CINAHL | 7,100 | [Edit](javascript:__doPostBack('ctl00$ctl00$MainContentArea$MainContentArea$editControl$printHistory$HistoryRepeater$ctl53$linkEditSearch','')) S9 |
| S8 | (MH "Community Health Nursing") OR "community health nursing" | Search modes - Boolean/Phrase | Interface - EBSCOhost Research Databases  Search Screen - Advanced Search  Database - CINAHL | 19,124 | [Edit](javascript:__doPostBack('ctl00$ctl00$MainContentArea$MainContentArea$editControl$printHistory$HistoryRepeater$ctl54$linkEditSearch','')) S8 |
| S7 | (MH "Community Health Services") OR "community health services" | Search modes - Boolean/Phrase | Interface - EBSCOhost Research Databases  Search Screen - Advanced Search  Database - CINAHL | 10,631 | [Edit](javascript:__doPostBack('ctl00$ctl00$MainContentArea$MainContentArea$editControl$printHistory$HistoryRepeater$ctl55$linkEditSearch','')) S7 |
| S6 | (MH "Physicians, Family") OR (MH "Primary Health Care") OR "physicians, primary care" | Search modes - Boolean/Phrase | Interface - EBSCOhost Research Databases  Search Screen - Advanced Search  Database - CINAHL | 34,250 | [Edit](javascript:__doPostBack('ctl00$ctl00$MainContentArea$MainContentArea$editControl$printHistory$HistoryRepeater$ctl56$linkEditSearch','')) S6 |
| S5 | (MH "Physicians, Family") OR "family physicians" | Search modes - Boolean/Phrase | Interface - EBSCOhost Research Databases  Search Screen - Advanced Search  Database - CINAHL | 9,135 | [Edit](javascript:__doPostBack('ctl00$ctl00$MainContentArea$MainContentArea$editControl$printHistory$HistoryRepeater$ctl57$linkEditSearch','')) S5 |
| S4 | (MH "Physicians, Family") OR "general practitioners" | Search modes - Boolean/Phrase | Interface - EBSCOhost Research Databases  Search Screen - Advanced Search  Database - CINAHL | 11,138 | [Edit](javascript:__doPostBack('ctl00$ctl00$MainContentArea$MainContentArea$editControl$printHistory$HistoryRepeater$ctl58$linkEditSearch','')) S4 |
| S3 | (MH "Primary Health Care") OR "Primary Health Care" | Search modes - Boolean/Phrase | Interface - EBSCOhost Research Databases  Search Screen - Advanced Search  Database - CINAHL | 29,955 | [Edit](javascript:__doPostBack('ctl00$ctl00$MainContentArea$MainContentArea$editControl$printHistory$HistoryRepeater$ctl59$linkEditSearch','')) S3 |
| S2 | (MH "Family Practice") OR "family practice" | Search modes - Boolean/Phrase | Interface - EBSCOhost Research Databases  Search Screen - Advanced Search  Database - CINAHL | 11,046 | [Edit](javascript:__doPostBack('ctl00$ctl00$MainContentArea$MainContentArea$editControl$printHistory$HistoryRepeater$ctl60$linkEditSearch','')) S2 |
| S1 | (MH "Family Practice") OR "general practice" | Search modes - Boolean/Phrase | Interface - EBSCOhost Research Databases  Search Screen - Advanced Search  Database - CINAHL | 12,893 | [Edit](javascript:__doPostBack('ctl00$ctl00$MainContentArea$MainContentArea$editControl$printHistory$HistoryRepeater$ctl61$linkEditSearch','')) |

**Search Date: 7^th^ October 2013**

| Medline | 885 |
| --- | --- |
| EMBASE | 1093 |
| PsycInfo | 304 |
| CINAHL | 530 |
| Duplicates | 1297 |
| All References | 2812 |
